# Prevalence of tobacco smoking among health-care physicians in Bahrain

**DOI:** 10.1186/1471-2458-14-931

**Published:** 2014-09-08

**Authors:** Saif M Borgan, Ghufran Jassim, Zaid A Marhoon, Mohamed A Almuqamam, Mohamed A Ebrahim, Peter A Soliman

**Affiliations:** Department of Family and Community Medicine, Royal College of Surgeons in Ireland- Medical University of Bahrain, P.O. Box 15503, Adliya, Kingdom of Bahrain

**Keywords:** Smoking, Physician, Waterpipe, Shisha, Cigarettes, Doctors, Tobacco, Lifestyle

## Abstract

**Background:**

There is a clear shift in smoking habits among the Middle Eastern population with a recent and alarming increase in the prevalence of waterpipe (shisha) smoking. This phenomenon has not yet been studied sufficiently across the physician population. Therefore, we set out to establish the smoking status of primary healthcare physicians in the kingdom of Bahrain.

**Methods:**

A self-administered questionnaire was distributed to a random sample of 175 out of the total 320 primary care physicians. Descriptive analysis was performed on all data and associations between variables were tested using Fishers Exact t test with statistical significance set as P-value < 0.05.

**Results:**

One hundred and fifty two physicians agreed to participate in the study. Sixty seven percent of physicians were females and the mean (SD) age was 45 (10) years. The majority of the physicians were married (93%) and of Bahraini nationality (76%). Ever-smokers were 11% of the population while current smokers corresponded to 8.6%. Waterpipe was the most common method of tobacco smoking followed by cigarettes. Among male physicians, the prevalence of current ‘waterpipe only’ smokers was 12%, followed by 4% and 2% corresponding to ‘cigarette only’ smokers and both, respectively. There were only three female smokers in the population, two ‘waterpipe only’ smokers and one cigar smoker. Of those who smoked waterpipe (n = 9; 6%), 33% smoked daily, 44% smoked weekly and 22% smoked at least once a month. Current smoking status was associated with male gender (P < 0.001) and showed a male to female smoking ratio of (10:1).

**Conclusion:**

Waterpipe smoking rates exceeded cigarette smoking among the population of physicians in Bahrain. Prevalence of smoking remains unacceptably high among male physicians. Assessment of physicians’ knowledge of the harmful effects of waterpipe tobacco smoking is warranted to plan future interventions.

## Background

According to the World Health Organization (WHO), tobacco smoking is the single largest preventable cause of death worldwide with an expected 1 billion smoking-related-deaths by the end of the 21st century [1]. Even though cigarettes remain the most common form of tobacco smoking, their prevalence has been slowly decreasing [2], while waterpipe use has been gaining popularity over the past two decades [3, 4].

Tobacco smoking constitutes a public health dilemma in the kingdom of Bahrain. The prevalence of cigarette and waterpipe smoking in 2007 was 13.8% and 8.4% respectively with the highest rates reported among males aged 20–29 years [5]. The Global Youth Tobacco Survey conducted in 2003 revealed smoking rates of 33.5% among males and 11.9% among females aged 13–15 years [6]. Recently, attempts were made to formulate new national guidelines on smoking cessation by integrating primary health care into smoking cessation services [7].

It is vital to assess health professionals’ smoking habits for two reasons. First, they have a direct effect on their health and wellbeing. Secondly, it has been shown that physicians who smoke tobacco are less likely to advise their patients regarding the health hazards of tobacco smoking [8–10]. Provided that previous studies have proven the effectiveness of physicians’ simple advice on smoking cessation rates among patients [11–14], reducing the smoking prevalence among physician may actually contribute to a reduction of the smoking prevalence among the general population. Furthermore, it has been shown that there is a relationship between physicians advice and smokers interest in quitting both; cigarettes [14] and waterpipe smoking [15].

One study (1999) conducted among primary health-care physicians in Bahrain reported that 26.6% of male physicians smoked tobacco, which is an alarming figure [16]. Another study, using a selective sample of physicians, reported smoking rates as high as 24.0% in 2005 [17]. It is important to monitor the change in smoking habits among the general population as well as individual populations such as primary health care physicians. Hence, we have conducted a cross sectional study to establish the prevalence and patterns of different methods of tobacco smoking among primary health care physicians in Bahrain.

## Methods

### Population and sampling

A list of all active full-time physicians was acquired from the ministry of health (N = 320). A computerized random sample generator was used to select a sample of 175 physicians who were distributed over all 27 primary healthcare service locations around the country. The sample size was estimated to yield at least a 90% confidence level of being within 5% of the true value, allowing for up to 20% of a non-response rate. From July to August 2013, physicians were approached during their allocated break time (10:30–11:00 am). We introduced them to the research and obtained their written consent. A self-administered confidential questionnaire in English was administered, and approximately 10 minutes were required to fill it in.

### Questionnaire

A descriptive questionnaire for the assessment of smoking habits among primary healthcare physicians was adapted from Al zayani [18]. The questionnaire specifically asked about cigarette smoking, waterpipe (shisha) and other forms of tobacco smoking. Former and never cigarette smokers were defined according to the Centre for Disease Control and Prevention (CDC) guidelines [19] and underlined under each question. Since there are no such guidelines for waterpipe smoking, we defined former waterpipe smokers as ‘used to smoke regularly at a frequency of at least once a month but have not smoked in more than one month’ and never waterpipe smokers as ‘I don’t smoke waterpipe and have never smoked waterpipe with a frequency of at least once a month or more’. Current and former smokers were asked about age of smoking initiation as well as age of quitting for the latter.

### Statistical analysis

Descriptive analysis was performed on all variables. Fishers’ exact t test was constructed to test associations between categorical variables. The statistical significance was defined as P <0.05.

### Ethical consideration

Ethical approval was obtained from the Royal College of Surgeons in Ireland-Bahrain Research Ethics Committee, The Ministry of Health (MOH) in Bahrain and the head of primary healthcare within the MOH. An information sheet as well as a consent sheet preceded each questionnaire and physicians were informed of the need for written consent.

## Results

### Population characteristics

One hundred and fifty two physicians practicing in all healthcare centers across Bahrain participated in the study. Table 1 shows demographics of the population including age, gender, nationality and marital status. Participants had a mean age of 45 (10) years with females comprising two thirds of the population.

**Table 1:** **Participants characteristics**

		n	%
Age	30-39	53	37
	40-49	42	29
	50-59	35	24
	> 60	15	10
Gender	Male	50	33
	Female	102	67
Nationality	Bahraini	117	77
	Non-Bahraini	35	23
Marital Status	Single	9	6
	Married	143	94
Specialization	General practitioner	34	23
	Family physician	116	77

### Smoking habits

The prevalence of tobacco smoking was 8.6% among all physicians (20.0% among males and 3.0% among females); 4.6% smoked waterpipe only (12.0% of males and 2.0% of females), 2% smoked cigarettes only (6% of males and 0% of females), 0.7% smoked waterpipe and cigarettes, 0.7% smoked waterpipe and Midwakh while 0.7% smoked Cigars only. Waterpipe was the predominant method of smoking among all primary care physicians with a mean initiation age of 27 years. The frequency of smoking indicated that 2.1% of the physicians smoked daily, 2.7% smoked weekly, and 1.4% smoked monthly. There were no former smokers of waterpipe tobacco in the sample (Table 2).

**Table 2:** **Prevalence of different smoking methods among male and female physicians**

		Gender			
		Male (n = 50)		Female (n = 98)	
		n	%	n	%
Cigarettes	Current smokers	4	(8)^a^	0	(0)
	Previous smoker	6	(12)	0	(0)
	Never smoker	40	(80)	98	(100)
Waterpipe (Shisha)	Current smoker	7	(15)^a^	2	(2)
	Previous smoker	0	(0)	0	(0)
	Never smoker	41	(85)	96	(98)
Other (Cigar/Midwakh)	Never smoker	49	(98)	101	(99)
	Current smoker	1	(2)	1	(1)

- One physician smokes both Waterpipe and Cigarette.

- One physician smokes both Waterpioe and Midwakh.

^a^Indicates a statistically significant difference between males and females (P < .05).

Current cigarette smokers consisted of 2.6% (8.0% males and 0% females) of the population, former cigarette smokers were 3.9% (12.0% males and 0% females) and never smokers were 89.5% (80.0% males and 94.1% females). Among current cigarette smokers, the mean initiation age was 22 years and the average number of cigarettes smoked per day was 15. The mean age of former cigarette smokers was 51 (9.6) years with a median smoking period of 14 years.

Among male physicians the prevalence of all forms of tobacco smoking was 20% compared with 3% among female physicians. Waterpipe and cigarette smoking were both associated with male gender, P = 0.006 and P = 0.012, respectively. Tobacco smoking was not associated with age, specialization, marital status or nationality.

## Discussion

It is evident that primary health care physicians play an important role in decreasing smoking rates among patients [20]. Further, their personal smoking habits have been shown to affect how frequently they provide cessation-related advice [8–10]. Our study has revealed that 20% of male physicians and 3% of female physicians are current smokers, and that waterpipe is the most common form of tobacco smoking among both genders. These results also suggest that the physician population is one of the first in which waterpipe smoking prevalence has surpassed that of cigarette smoking.

The rates of smoking among male physicians were alarmingly high when compared with male physicians in Canada (8%) [21], Switzerland (12.6%) [22] and Japan (16.2%) [23]. However, observed differences might be affected by the definition of a smoker and distribution of gender and age among physicians. For example, in the Swiss study [21], smokers and former smokers were defined according to whether they smoked one cigarette daily while in our study we considered five packs or 100 cigarettes smoked in the past as the cut off point for former smokers as per CDC guidelines [17]. Additionally, there was a male predominance of 84% and a relatively older age compared with the population in our study (51 vs 45 years of age). In comparison with the Bahraini general population [5], the prevalence of current smokers of all forms was less (9% vs 19.9%). However, the rate of waterpipe smoking among male physicians was higher than in male adults in the general population (12% vs 10.8%) Table 3.

**Table 3:** **Comparison of smoking prevalence between physicians and the general population (%)**

	General population 2010 [5]			Physicians 2013 ^b^		
	Male	Female	Total	Males	Female	Total
Cigarette	27	1	13	8	0	3
Waterpipe	11	6	8	15	2	6

^b^Current study.

Former studies conducted among primary care physicians in Bahrain [16, 17] found a higher prevalence of male smokers (26.6% in 1999, 24% in 2005 and 20% in the current study), but they applied different methodologies compared with the current study. For example, the study conducted in 2005 did not define ‘smoker’ and used a selective sample as their population while in the study in 1999, ‘smoker’ was defined differently than in this study. However, our results revealed a higher percentage of waterpipe smokers (<1% in the 1998 study, 3% in the 2005 study, and 6% in the current study). The increasing use of waterpipes among physicians as well as the general population could be attributed to various reasons, including the common misconception that waterpipe smoking is less harmful than cigarette smoking [4], the introduction of flavoured tobacco to attract waterpipe users [24] and flattering images in the media of waterpipe tobacco smoking [25]. Therefore, our results have direct implications for necessary public health interventions targeting primary care physicians. Additionally, an attempt to reverse the current smoking patterns should be made by increasing awareness of the harmful effects associated with any method of tobacco smoking including waterpipe smoking and promoting legislation to remove all waterpipe tobacco flavours.

The high male predominance of smokers among our population, which is consistent with the previous study [16], could be attributed to cultural factors with a higher perceived stigma among females who smoke [26, 27]. This phenomenon has been observed to a lower extent for waterpipe smoking compared with cigarette smoking, and may potentially explain the smoking habits of female physicians in our sample (0% cigarette smokers compared with 2% waterpipe smokers).

Further research of physicians’ habits, attitudes and knowledge regarding waterpipe tobacco smoking has been gaining more attention because the role of the physician in influencing smoking cessation among their patients has been recognized. Furthermore, The WHO, CDC, and the Canadian Public Health Association have developed the Global Health Professional Survey to collect data on tobacco use and cessation counselling among health professional students. Physicians’ knowledge and attitudes towards waterpipe tobacco smoking and their related cessation advice practices have not yet been measured in Bahrain and represent a potential area for future research.

Limitations of our study include non-response bias. Non-responders, comprising 15% of the current sample could have affected the rates of smoking, which could have been higher, especially among female smokers who may have preferred to refrain from answering the questionnaire. This may have led to an underestimation of smoking prevalence. Another limitation is that we only studied smoking prevalence and did not look into perceptions and knowledge regarding waterpipe and cigarette smoking which could have given us more insight into conducting future interventions.

Educating patients and increasing awareness of the harmful effects of cigarette and waterpipe smoking are the responsibility of all physicians. By encouraging physicians to emulate the positive lifestyle choices they recommend, they can become better equipped to convince their patients to make real changes in their lives. This holds great potential and opportunity to make measurable changes in the prevalence of smoking in Bahrain.

## Conclusion

There is a high prevalence of tobacco smoking among primary health care physicians in Bahrain. We have established that there has been a small decline in cigarette smoking since 1999 and that waterpipe is, currently, and for the first time, the most common form of tobacco smoking among physicians. We have also shown that there is a clear male predominance in smoking, consistent with the prevalence among the general population and previous studies conducted on primary care physicians.

## Authors’ information

This work was completed by medical students at the Royal College of Surgeons in Ireland – Bahrain and supervised by senior lecturer; Dr. Ghufran Jassim, as part of RCSI-Student Summer Research Programme.

## Abbreviations

CDC: Center for Disease Control and Prevention
WHO: World Health Organization
CPHA: Canadian Public Health Association
GHPS: Global Health Professional Survey.
